# Relating Phage Genomes to *Helicobacter pylori* Population Structure: General Steps Using Whole-Genome Sequencing Data

**DOI:** 10.3390/ijms19071831

**Published:** 2018-06-21

**Authors:** Filipa F. Vale, Philippe Lehours

**Affiliations:** 1 Host-Pathogen Interactions Unit, Research Institute for Medicines (iMed-ULisboa), Faculdade de Farmácia, Universidade de Lisboa, 1649-003 Lisboa, Portugal; 2 Laboratoire de Bacteriologie, Centre National de Référence des Campylobacters et Hélicobacters, Place Amélie Raba Léon, 33076 Bordeaux, France; 3 INSERM U1053-UMR Bordeaux Research in Translational Oncology, BaRITOn, 33000 Bordeaux, France

**Keywords:** phage, genome, phylogeography, *Helicobacter pylori*, evolution

## Abstract

The review uses the *Helicobacter pylori*, the gastric bacterium that colonizes the human stomach, to address how to obtain information from bacterial genomes about prophage biology. In a time of continuous growing number of genomes available, this review provides tools to explore genomes for prophage presence, or other mobile genetic elements and virulence factors. The review starts by covering the genetic diversity of *H. pylori* and then moves to the biologic basis and the bioinformatics approaches used for studding the *H. pylori* phage biology from their genomes and how this is related with the bacterial population structure. Aspects concerning *H. pylori* prophage biology, evolution and phylogeography are discussed.

## 1. Introduction

*H. pylori* is a Gram-negative bacterium that colonizes about half of the human population and is associated with several gastrointestinal diseases, such as gastritis (all cases), peptic ulcer (20% of the infected individuals), and in rare cases gastric cancer (1%) and gastric MALT (Mucosa Associated Lymphoid Tissue) lymphoma (<1%) [1]. *H. pylori* shares a co-evolutionary history with the human host presenting a similar phylogeographic structure, which allows the reconstruction of human migrations [2,3]. *H. pylori* is characterized by its high genome diversity attributed to high mutation and recombination rates [4,5]. *H. pylori* presents an extraordinary diversity and high number of restriction-modification systems [6,7], which appear to inhibit import of heterologous DNA, but not homeologous recombination [8] that is very frequent in *H. pylori* [9]. Another source of genetic diversity in *H. pylori* arrives from mobile genomic elements, that present their own characteristic phylogeographic signal [10,11]. Prophages are widespread in the bacterial world and can be transferred to new hosts by horizontal gene transfer, which has been suggested to be pervasive in natural bacterial populations [12]. The aim of this review is to revisit *H. pylori* prophages and to provide tools for their study, especially focusing on how phage genomes are related with the bacterial population structure.

## 2. *H. pylori* Population Structure and Human Migrations

**Figure 1:**
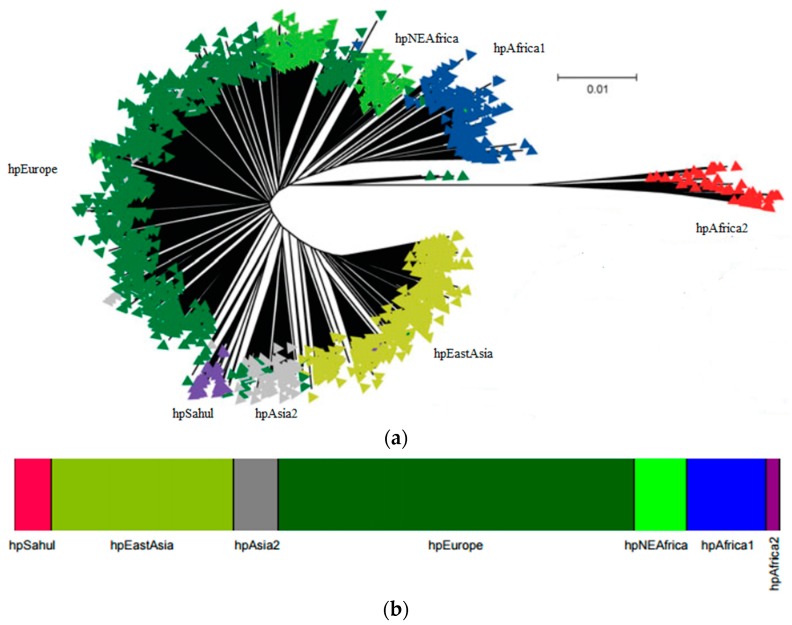
Worldwide population structure of *H. pylori*: (**a**) Neighbor-joining tree using Kimura two-parameter model of the concatenated *H. pylori* housekeeping genes (adapted with permission from [22]); (**b**) DISTRUCT plot of the Bayesian assignment of *H. pylori* to populations using STRUCTURE V2.0 with no admixture model, where each isolate is represented by a thin line that is color coded according to the population assignment (adapted with permission from [3]).

The study of seven housekeeping genes of *H. pylori* has been widely used to characterize the strains. The genes used for genes multilocus sequence typing (MLST) are *atp*A, *efp*, *mut*Y, *ppa*, *trp*C, *ure*A and *yph*C [13]. The Bayesian clustering of these seven housekeeping genes applied to hundreds of strains from distinct geographic regions [2,14] revealed the presence of seven modern populations of *H. pylori* that clusters according to the geographic origin of the bacterium and its host (reviewed in [15,16,17]). The seven modern populations of *H. pylori*, hpAfrica2, hpAfrica1, hpNEAfrica, hpSahul, hpAsia2, hpEurope and hpEastAsia (Figure 1a,b), evidences that *H. pylori* and man co-evolved together, since they went “out of Africa” [2,3,14]. Each of these populations may be divided into subpopulations. For example hpAfrica1 is currently divided in hspSAfrica, hspWAfrica and hspCAfrica. The structured population provided strong evidence of ancient ancestry in Africa and of co-evolution with the human host since then. The original Human migration from Africa to the Middle East is estimated to have occurred ~60,000–150,000 years ago and then independently to Europe and Asia [18,19,20].

*H. pylori* is a highly recombinogenic species [21]. Considering that recombination requires physical exchange of genomic DNA, recombination is more evident within populations than among populations [22]. While mutations are passed vertically to the offspring, recombination occurs between unrelated organisms that can create homoplasies, i.e., a similar sequence acquired from an unrelated lineage. This form of convergent evolution may biases the reconstructions of clonal phylogenies. This effect is observable in Figure 1a where branches separating strains are much longer than the ones separating populations [22]. Figure 1b shows the resulting population assigned using the number of bacterial populations (K = 7) using the program STRUCTURE, that uses a Bayesian approach. This program is run for several values of K and in each run, for each K, a Markov Chain Monte Carlo simulation of thousand of iterations approximate the posterior probability of K. The number of populations (K) that best clusters the data presents simultaneity higher posterior probability and is biologically interesting, i.e., correspond to real populations [23].

Next Generation Sequencing (NGS) is accelerating biological research in many areas such as genomics, transcriptomics, metagenomics, proteogenomics, gene expression analysis, noncoding RNA discovery, Single Nucleotide Polymorphism (SNP) detection, identification of protein binding sites, among others [24,25]. The increasing number of *H. pylori* genomes available provides a mean to obtain more information about its phylogeny. This is the case of the overcome of the difficulty in inferring the population structure due to high recombination rate found in *H. pylori*. Briefly, a method called chromosome painting in silico [26] was used to detect the transfer of DNA sequence chunks between genomes through homologous recombination throughout the genome [5]. A co-ancestry matrix is generated showing the expected number of chromosome chunks imported from a donor to a recipient genome. The matrix is then used to assign each strain to a subgroup using fineSTRUCTURE clustering algorithm [26]. This method revealed a finer population structure than the one based on the genes used by MLST typing [5,27].

### Ancestral H. pylori Populations

**Figure 2:**
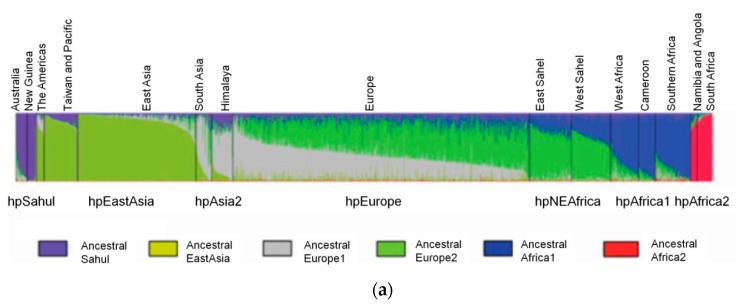

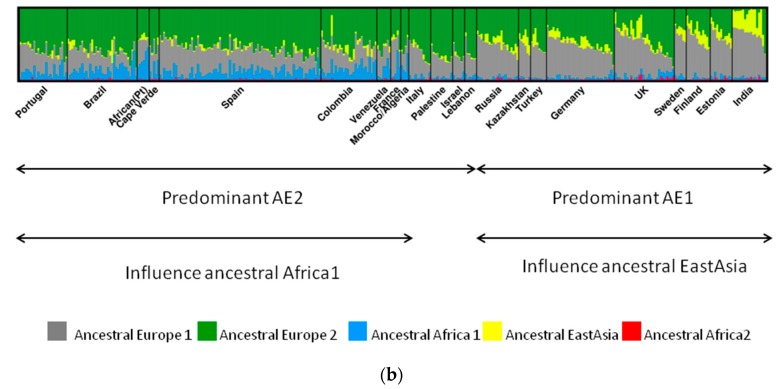
Ancestral populations of *H. pylori*. Each vertical line represents a strain. (**a**). General 6 ancestral populations of *H. pylori*. Modern *H. pylori* populations result from the admixture of ancestral populations (adapted with permission from [22]); (**b**). Detailed ancestral populations found in Europe. All hpEurope strains are recombinants of AE1 and AE2, but southwest Europe strains are additionally a product of recombination with ancestral Africa1 (adapted with permission from [29]).

The STRUCTURE software has three model options, the “no admixture model”, the “admixture model” and the “linkage model”. The selection of the most appropriate model depends on the user’s data and study objectives. The “no admixture model” is the simplest case where each individual is assumed to have originated in a single population, whereas when there is prior knowledge about the origin of the populations under study and there is no reason to consider each population as completely discrete, the “admixture model” is appropriate. The “linkage model” is like the admixture model, but linked loci are more likely to come from the same population. The linkage model relies on linkage disequilibrium—the nonrandom association of alleles at different loci—that is a sensitive indicator of the population genetic forces that structure a genome [23]. There are currently six ancestral or precursor populations inferred to *H. pylori* using the linkage model of STRUCTURE (Figure 2) to analyze the seven housekeeping genes used for MLST. These are Ancestral Sahul, Ancestral EastAsia, Ancestral Europe 1 (AE1), Ancestral Europe 2 (AE2), Ancestral Africa1 and Ancestral Africa2 [2,22,28]. Modern populations were produced by admixture of ancient populations.

The case of hpEurope is particularly interesting, as this population is a recombinant of mainly AE1 and AE2 [2]. AE1 probably entered Europe via central or southern Asia, while AE2 entered Europe via Northeast Africa or Southern Europe [2,29]. The strains from India assigned to hpEurope revealed residual evidence of AE2, but presented a higher influence of ancestral EastAsia (Figure 2). This influence of ancestral EastAsia was still observed, even that in a small scale, for countries where AE1 is more predominant, favoring the entering of AE1 through Asia. Southern European countries presented a higher proportion of AE2 (Figure 2). Interestingly, Iberian countries also present influence of the recombination with ancestral Africa1, that is even higher than AE1 in a few hpEurope strains from African Portuguese speaking countries [29].

The spread of AE2 to Europe may have occurred during the Paleolithic population expansion from the “Atlantic zone” (southwestern Europe) 10,000–15,000 years ago, after the Last Glacial Maximum [30]. It is also feasible that a second wave of migration from Africa to Iberia during the Arab Empire (711–1249) introduced ancestral Africa1. During the Arab empire the Iberia peninsula colonizers were mainly Berbers from North Africa, and not Arabs, which is in agreement with ancestral proportions of each population found in Iberian countries and northern Africa (AE1, AE2 and ancestral Africa1) and middle east (AE1 and AE2, but not ancestral Africa1). Before this period there was a commercial trade between Iberia peninsula and Mediterranean nations, which also may explain the influence of ancestral Africa1 [29]. The exact way AE1 and AE2 recombination occurred is controversial, but may arose latter than previously expected, since the 5000 years old Iceman mummy found in Italian border presented only AE1 ancestry [31].

## 3. Bacteriophages

Bacteriophages may present a lytic or lysogenic life cycle. The former lyse the bacterial host cell after viral replication, allowing the release of newly formed phage particles. The latter constitute lysogenic or temperate phages, which are able to switch between lytic and lysogenic cycles. If pursuing the lysogenic cycle, the phage genome is integrated in the bacterial genome and gains the designation of prophage. These continuous process of phage insertion and excision from the bacterial host genome can provide a mean of changing various genes among bacteria, some of which may provide an advantage to the host cell, for instance promoting antibiotic resistance or virulence [32]. Phage integrases and excisionases mediate integration and excision from the host cell genome at specific attachment sites of bacteria (attB) and phage (attP) genomes, respectively [33]. Another less frequent phage life cycle is pseudolysogeny, described as an unstable situation in which the phage genome fails to replicate (lytic cycle) or become established as a prophage (lysogenic cycle). Pseudolysogeny has been associated with nutrient-deprived conditions, that impairs DNA replication or protein synthesis, during which the phage genome remains for an extended period of time as a non-integrated preprophage, similar to an episome [33,34]. According to this hypothesis when the nutritional status is restored the phage enters either a lysogenic or a lytic life cycle [33].

Despite the putative bacterium–phage evolutionary conflict, phages profit from promoting the survival and proliferation of their hosts [33]. Likewise, prophages may harbor cargo genes, or “morons”, which while are not essential for the phage, benefits the host. Some very well known lysogenic phages carry genes that enhance the virulence of the bacterial host [35]. In addition, the deletion of prophages from *Escherichia coli* revealed that prophages improved the surviving under adverse environmental conditions, including acid stress or early biofilm formation [36]. Prophages may therefore work as gene reservoirs, many of which benefit pathogens, in ways which are only just beginning to be determined [37]. In a hostile environment, such as the human stomach, any metabolic advantage or resistance/tolerance mechanism provided by prophages should be important in improving bacterial host competitiveness. Prophage induction may also be used as a weapon for colonizing new niches [38], displacing native strains, although this strategy may be rarely used, first by the creation of lysogens in the susceptible population, second by the cost of cell lysis in a fraction of the population, and third due to the purifying selection of prophages [39]. Taken together, these properties may explain why prophages are more frequent in pathogenic bacteria [40]. Host-prophage driven selection and genetic flux occurs even for prophage genes that do not effect host physiology [39]. Thus, the role of prophages in disease establishment is being progressively acknowledged.

### 3.1. H. pylori Phages and Prophages

**Figure 3:**
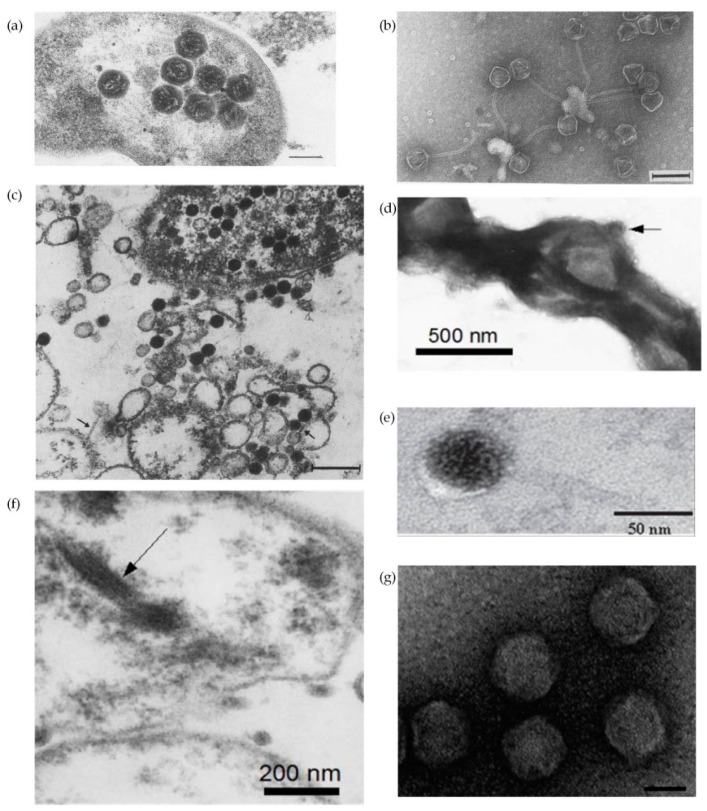
Electron micrographs of *H. pylori* phages: (**a**) Thin section of *H. pylori* carrying phage particles with phage head diameter of about 85 nm. Bar = 100 nm (adapted with permission from [53]); (**b**) Negative staining of *H. pylori* phage HP1. Phage head diameter of about 50 to 60 nm and tail with 170 nm and a diameter of 9.5 nm. Bar = 100 nm (adapted with permission from [56]); (**c**) Thin section of *H. pylori* evidencing cell with empty and filled phage heads. Phage head diameter with (70 ± 5) × (60 ± 4) nm and tail with 120 nm long. Arrows point phage tails in extracellular phages. Bar = 200 nm (adapted with permission from [55]); (**d**) Negative staining of *H. pylori* cell with arrow pointing to a polyhedral phage-like particle with a diameter of about 100 nm without tail (adapted with permission from [54]); (**e**) Thin section of *H. pylori* phage phiHP33, presenting a total length of 150 nm, a phage head diameter of 62.5 nm (±7.3 nm), and a tail with 92.4 nm (±2.97 nm) long and 5 to 6 nm in diameter (adapted with permission from [58]); (**f**) Arrow points to hypothetical 15 nm phage filamentous in *H. pylori* cell (adapted with permission from [54]); (**g**) Negative staining of intact *H. pylori* KHP30 phage particles with no tail and head diameter of 68.8 nm (±2.3 nm). Bar = 50 nm (adapted with permission from [61]).

One of the remarkable characteristics of *H. pylori* is the extensive genetic diversity between different strains [6,7,14,41,42]. This diversity has been attributed to an elevated high mutation rate, impaired DNA repair, lateral DNA transfer and frequent recombination events [43]. Horizontal gene transfer, the movement of genetic material between different genomes, constitutes a key evolutionary force that shapes bacterial genomes and may contribute to niche adaptation through gaining of genes that provide selective advantages [44,45]. Importantly, horizontal gene transfer plays a role in spreading antibiotic resistance [46,47]. Plasmids, transposons and phages are mobile genetic elements that mediate horizontal gene transfer, all of them known to be present in *H. pylori*. The horizontal gene transfer may be mediated by transformation (transfer of a naked DNA fragment), conjugation (direct transfer between two bacteria temporarily in physical contact) and transduction (transport of bacterial DNA by phages), but also by membrane vesicles and autolysis [44]. Conjugation [43,48] and transformation [49,50] have already been described for *H. pylori*, but not transduction. There are about 10^31^ phages on the planet, with phages exceeding bacteria in number by tenfold, but less than an estimated 1% have been described [51]. Bacteriophage description in *H. pylori* is brief in the literature. The first descriptions of *H. pylori* phages came from the observation of micrographs where particles compatible with phages were observed (Figure 3) [52,53,54,55,56]. All but Figure 3f appear to be icosaedric phage particles, which typically have an icosahedral capsid protein and a double stranded DNA genome. Based on their morphology only phages in Figure 3a,d,g are compatible with *Corticoviridae* and *Tectiviridae*, without tail but with a lipidic content; phages in Figure 3b,c,e show similarities with family *Siphoviridae*, having a long non-contractil tail; while phages in Figure 3f, considering the morphological filamentous form, are similar to *Inoviridae*, which harbor single stranded DNA small genomes. However, the nucleic acid of the filamentous phage present in Figure 3f was not isolated.

The development of the genomic studies, especially using high-throughput genome sequencing led to the first reports of prophages, some remnant [57], others apparently complete and capable of going through a lytic cycle [58,59,60,61,62]. A screening for prophages in public available genomes of *H. pylori* revealed the presence of prophage sequences ranging from 5.5 to 39.3 Kb [63]. Table 1 offers a compendium of *H. pylori* phage particles identified so far. Strains carrying prophages do not appear to have a higher pathogenicity or association with particular disease patterns [10,58], but it has been suggested that the presence of phage orthologous genes correlates with the presence of *cagA* and/or *vacA* virulence genes [64]. Despite the putative bacterium–phage evolutionary conflict, phages profit from promoting the survival and proliferation of their hosts [33].

The intricate and complex co-evolutionary process shared by bacteria and their viruses is difficult to ascertain [51]. There are several evidences of this co-evolutionary process, some of which are: (i) phylogenetic agreement between integrase phage gene and MLST genes, both showing a similar phylogeographic segregation, although the existence of some differences; (ii) the probable acquisition of prophages before *Helicobacter* speciation, evidenced by the existence of prophage genes in other *Helicobacter* species, like *Helicobacter acinonychis* [65], *Helicobacter felis* [66], or *Helicobacter bizzozeronii* [67]; (iii) a similar genetic syntheny of phage genes of distinct lineages [58]; (iv) and, finally, the occurrence of prophage remnants in both *H. pylori* [68,69] and *non-pylori* Helicobacters, [70] suggesting phage inactivation by an ongoing phage decay process. It is feasible to accept that at any point a strain may be infected by a phage from a particular lineage, starting at this moment the interaction between phage and bacteria, meaning that the observation of a prophage in a genome does not offer information about how long it has been there. Nonetheless, the points highlighted above strongly suggest a prolonged co-evolutionary history [10].

### 3.2. Contribution of Prophages Genomes to H. pylori Population Structure

**Figure 4:**
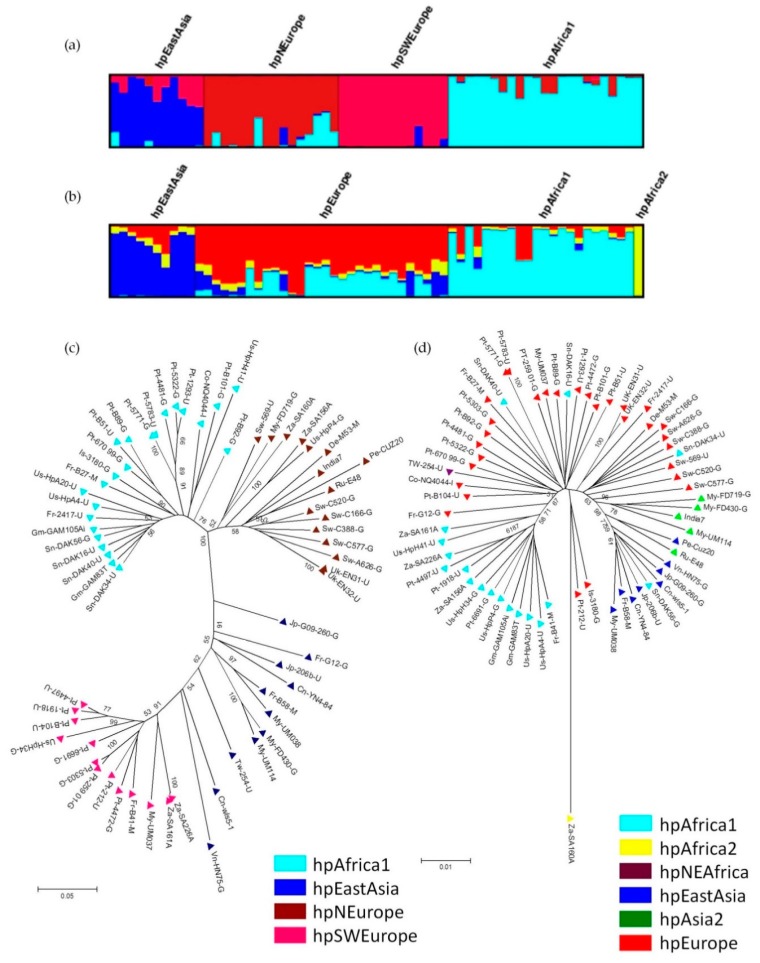
Population structure of *H. pylori* prophages: (**a**) DISTRUCT plot of Bayesian population assignments using STRUCTURE and an admixture model (K = 4) for prophages genes (PST); (**b**) Same methodology used for the sequences of seven housekeeping genes (MLST). Each bacterial isolate is depicted by a thin vertical line, which is divided into K colored segments representing the membership coefficients in each cluster; (**c**) Neighbour-joining tree (Kimura 2-parameter) of concatenated prophage sequences; (**d**) Neighbour-joining tree (Kimura 2-parameter) of concatenated sequences of MLST genes. In C and D the strains are colour-coded according to the population assignment by STRUCTURE using PST and MLST genes, respectively (adapted from [10]).

Interestingly, like their host, *H. pylori* prophages also present a phylogeographic distribution. The population to which prophages belong was determined by prophage sequence typing (PST), which targets two prophage genes (integrase and holin) of *H. pylori* and applies a Bayesian clustering analysis for the identification of distinct genetic populations. The prophage genes used by the PST method are the integrase (responsible for the integration of the phage genome into the bacterial chromosome) and holin (involved in cell lysis when a lytic cycle occurs). Currently there are 4 prophage populations described (Figure 4), hpAfrica1, hpEastAsia, hpNEurope and hpSWEurope [10,71].

### 3.3. Phage Detection and Annotation

Identification of phage genes is similar to any other gene annotation process. There are several annotation pipelines [72], such as RAST [73], Prokka [74] and others. Genome annotations starts by identifying genes, or more precisely open reading frames (ORF), i.e., identifying start and stop positions in same frame of the prokaryote genome, along with function identification. Predicting ORF is done using software like Glimmer [75] or GeneMark [76]. The next step consists in using these predictions and search databases, such as Genbank [77] and SwissProt [78], using mainly BLAST [79], or other programs. The accuracy of this step depends of the annotation software and the quality of the annotations already in the reference database [72,80]. To efficiently decrease annotation error comparison of results from multiple annotation services should be performed, interchanging information between annotation services [80]. There are, however, specific packages and web servers for detecting and inferring prophage presence and completeness. Search for homologous may be done in relevant protein viral databases, like Phantome (http://www.phantome.org/), PHAST/PHASTER database [81], and VirSorter [82]. These packages include the first ones developed, namely Prophage Finder [83], Phage_Finder [84] and Prophinder [85]. Recently PHAST and PHASTER were developed performing 40 times faster and presenting results up to 15% more sensitive in comparison with the previous ones [81,86].

Other prophage identification tools have been described, such as PhiSpy [87] which analyzes several other sequence-based statistics to help identify novel phages (AT and GC skew, protein length and transcription strand directionality) that are not represented in existing phage databases, and VirSorter [82] that handles metagenomic data with improved performance for fragmented genomes. These tools may run locally like (PhiSpy, Phage_Finder, Prophage Finder), to access through a web-server (PHAST/PHASTER), or made available through cyberinfrastructure (iPlant Discovery Environment [88], a Web portal of iPlant’s cyberinfrastructure that houses several apps for sequencing analysis and other data-intensive technologies) that provides a web-based user interface. The strategies applied by the above phage finder tools are based on databases of gene and protein sequences of phage origin and other typical sequences found in phages, such as attachment sites *attP* and *attB*. Thus, finding novel prophages sequences is challenging when there is no counterpart in the database that serves the tool. Moreover, these tools allow to identify the contigs presenting phage sequences, but not necessary the complete phage sequence, which is rarely present in one contig only [89]. To overcome these limitations an additional step using BLAST with a query of a complete nucleotide phage sequence to check for homologies in the contigs is helpful. The BLAST analysis is not only useful to confirm the presence of a phage sequence, but also to determine the order of the contigs in the phage genome. Based on this predicted contig order, primers flanking the contigs can be designed to bridge and close the gaps in the assembly.

Assembled prophages should then be annotated using general annotation pipelines, or specific for phage annotation, such as PHAST [81], Phages v. 1.0 (available online: http://www.phantome.org/PhageSeed/Phage.cgi?page=phast). Phage genomes typically have mosaic architectures and contain several small open reading frames of unknown function, approximately two-thirds the average size of bacterial genes, which is challenging for annotation and comparative analysis [90].

Available tools to predict the potential gene function include BLASTP (using local or NCBI databases) [79], HHPred (more sensitive then BLASTP and allowing protein structure prediction) [91], presence of conserved domains detected using BLAST, and pairwise comparisons of protein-coding genes to group genes by sequence similarity or conserved domains [92]. Other important aspect to assign gene function is genome synteny, since many of the genes appear in the same order and grouped by function. The gene functions of phage genes can be grouped in structure and assemble, DNA replication, life cycle regulation, cell lysis and other well characterized genes, like toxin/anti-toxin genes. Nevertheless, false positive identification cannot be ruled out and different approaches should be considered [90], keeping in mind that wet-lab experiments are the gold-standard.

Other available tools allow to predict the phage life cycle according to the protein function [93]. While integrase and excision genes favor the attribution of lysogenic cycle, the nature of structural proteins favors the identification of lytic cycle [93]. Using this tool, considering *H. pylori* phage genomes, all were confidently predicted as having a temperate life cycle. Other comparative genomics studies follow the general guidelines introduced above.

### 3.4. Potential Use of Phages to Eradicate H. pylori

The use of phages to deal with bacterial infections is older than the introduction of antibiotics, whose discovery limited the use and investigation in the field of phage therapy. Worldwide antibiotic resistance is increasing treatment challenges in general for bacteria and particularly for Gram-negatives like *H. pylori*. This global antibiotic crisis was recognized by the World Health Organization (WHO) that published in 2017 the first ever list of antibiotic-resistant priority pathogens for research and development of new antibiotics [94]. The list is mostly constituted by multi-resistant Gram-negative pathogens, including *H. pylori*, for which no new antibiotic class active can be anticipated in the near future. Phage therapy use lytic phages or their lytic lysins, to specifically treat infectious diseases caused by the phage host bacterium [95]. Phage therapy in its 100 years anniversary in light of its capability to kill susceptible organisms has attracted much attention as potential substitute for conventional antibiotics [96]. Phage therapy studies focusing *H. pylori* are a missing topic in the literature, but the continuous effort of fundamental research to describe and comprehend their gene function and role in phage-host co-evolution introduces the seed for future studies.

## 4. Conclusions

*H. pylori*, through the bacterium phylogeographic distribution, continues to provide insight about human migrations and admixture of populations, initially using Sanger sequencing of housekeeping and presently NGS technology. The later using the whole genome provides in-depth characterization of the population structure at finer level. Additionally, information from prophages genomes is related with the bacterial host population structure and may provide additionally information about subpopulations. In a time of continuous growing number of genomes available, the tools available for phage detection are important to find and characterize phages. Some of these tools can be adapted to explore genomes for other mobile genetic elements or virulence factors. Moreover, *H. pylori* phages may in the future be helpful to treat *H. pylori* infection.

## Figures and Tables

**Table 1:** Characteristics of *H. pylori* phages particles.

Name	Family	Gene Number	Genome Size (kb)	Gene Functions	Head/Tail Size (nm)	Reference
HP1	*Siphoviridae*	nd	22	nd	50–60/170 × 9.5	[56]
phiHP33	*Siphoviridae*	27	24.6	Integration	55–70/92 × 6	[58]
				regulation, replication, structural, lysis		
KHP30	*Corticoviridae*/*Tectiviridae* *	30	26.2	Integration	67–71/absent	[61]
				replication, structural, lysis		
1961P	*Podoviridae*	33	26.8	Integration	68–74/23 × 13.3	[59]
				replication, structural, lysis		

* but with larger genome size and (pseudo)lysogenic cycle; nd—not determined.

## Abbreviations

MALT	Mucosa Associated Lymphoid Tissue
MLST	Multilocus Sequence Typing
NGS	Next Generation Sequencing
ORF	Open Reading Frame
PST	Prophage Sequence Typing
SNP	Single Nucleotide Polymorphism
